# 2,7-Bis(prop-2-yn-1-yl­oxy)naphthalene

**DOI:** 10.1107/S1600536810020982

**Published:** 2010-06-09

**Authors:** Wei-jian Xue, Hui Lu, Tao Pang

**Affiliations:** a Key Laboratory of Pesticides and Chemical Biology of the Ministry of Education, College of Chemistry, Central China Normal University, Wuhan 430079, People’s Republic of China

## Abstract

The title compound, C_16_H_12_O_2_, was synthesized from naphthalene-2,7-diol and prop-2-ynyl 4-methyl­benzene­sulfonate in the presence of sodium hydride. The crystal packing exhibits inter­molecular non-classical C—H⋯O hydrogen bonds and C—H⋯π inter­actions.

## Related literature

For the preparation of the title compound, see: Srinivasan *et al.* (2006 ▶).
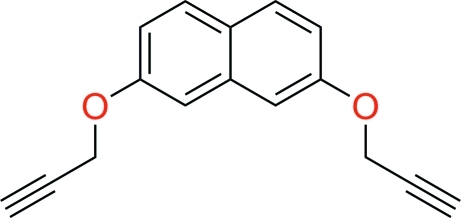


## Experimental

### 

#### Crystal data

C_16_H_12_O_2_
*M* *_r_* = 236.26
Orthorhombic, 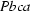

*a* = 11.3742 (12) Å
*b* = 8.1364 (9) Å
*c* = 26.880 (3) Å
*V* = 2487.6 (5) Å^3^
*Z* = 8
Mo *K*α radiation
μ = 0.08 mm^−1^
*T* = 298 K
0.16 × 0.12 × 0.04 mm

#### Data collection

Bruker SMART 1000 diffractometer
8492 measured reflections
2812 independent reflections
1807 reflections with *I* > 2σ(*I*)
*R*_int_ = 0.070

#### Refinement

*R*[*F* ^2^ > 2σ(*F* ^2^)] = 0.063
*wR*(*F* ^2^) = 0.146
*S* = 1.02
2812 reflections
163 parameters
H-atom parameters constrained
Δρ_max_ = 0.17 e Å^−3^
Δρ_min_ = −0.23 e Å^−3^

### 

Data collection: *SMART* (Bruker, 1999 ▶); cell refinement: *SAINT* (Bruker, 1999 ▶); data reduction: *SAINT*; program(s) used to solve structure: *SHELXS97* (Sheldrick, 2008 ▶); program(s) used to refine structure: *SHELXL97* (Sheldrick, 2008 ▶); molecular graphics: *SHELXTL* (Sheldrick, 2008 ▶); software used to prepare material for publication: *SHELXTL*.

## Supplementary Material

Crystal structure: contains datablocks I, global. DOI: 10.1107/S1600536810020982/om2339sup1.cif

Structure factors: contains datablocks I. DOI: 10.1107/S1600536810020982/om2339Isup2.hkl

Additional supplementary materials:  crystallographic information; 3D view; checkCIF report

## Figures and Tables

**Table 1:** Hydrogen-bond geometry (Å, °) *Cg*2 is the centroid of the C4–C9 benzene ring.

*D*—H⋯*A*	*D*—H	H⋯*A*	*D*⋯*A*	*D*—H⋯*A*
C13—H13⋯O2^i^	0.93	2.54	3.465 (3)	172
C14—H14*B*⋯O1^ii^	0.97	2.57	3.533 (3)	173
C11—H11*A*⋯*Cg*2^ii^	0.97	2.67	3.526 (3)	148

Symmetry codes: (i) 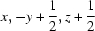; (ii) 
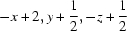
.

**Fig. 1.**
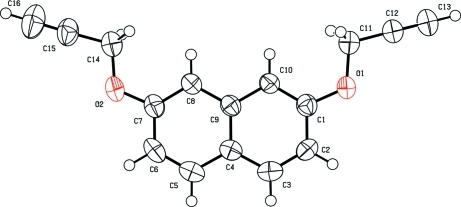
View of the title compound showing the atom-labelling scheme. Displacement ellipsoids are drawn at the 30% probability level. H atoms are represented by spheres of arbitrary radius.

## supplementary crystallographic information

### Comment

Naphthalene derivatives have manifested applications in many fields, for example, as a colorant, explosive, disinfectant, insecticide and plant hormone auxin. The reaction which between hydroxybenzene and prop-2-yn-1-yl 4-methylbenzenesulfonate gives rise to the product with rapid reaction rates by the introduction of sodium hydride (Srinivasan *et al.*, 2006;).

Here we report the crystal structure of the title compound (Fig. 1). The crystal packing exhibits non-classical C—H···O hydrogen bonds and C—H···π interaction (Table 1).

### Experimental

The title compound was synthesized according to the literature procedure of Srinivasan *et al.* (2006). Single crystals suitable for X-ray diffraction were prepared by slow evaporation of a solution of the title compound in chloroform : methanol (10:1) at room temperature.

### Refinement

All H atoms were initially located in a difference map, but were constrained to an idealized geometry. Constrained bond lengths and isotropic displacement parameters: (C—H = 0.97 Å) and *U*_iso_(H) = 1.2 *U*_eq_(C) for methylene, (C—H = 0.93 Å) and *U*_iso_(H) =1.2*U*_eq_(C) for aromatic H atoms, and (C—H = 0.93 Å) and *U*_iso_(H) = 1.2*U*_eq_(C) for alkynyl aromatic H atoms.

### Crystal data

**Table:**

C_16_H_12_O_2_	*F*(000) = 992
*M**_r_* = 236.26	*D*_x_ = 1.262 Mg m^−^^3^
Orthorhombic, *P**b**c**a*	Mo *K*α radiation, λ = 0.71073 Å
Hall symbol: -P 2ac 2ab	Cell parameters from 1273 reflections
*a* = 11.3742 (12) Å	θ = 3.0–22.2°
*b* = 8.1364 (9) Å	µ = 0.08 mm^−^^1^
*c* = 26.880 (3) Å	*T* = 298 K
*V* = 2487.6 (5) Å^3^	Plate, colorless
*Z* = 8	0.16 × 0.12 × 0.04 mm

### Data collection

**Table:**

Bruker SMART 1000 diffractometer	1807 reflections with *I* > 2σ(*I*)
Radiation source: fine-focus sealed tube	*R*_int_ = 0.070
graphite	θ_max_ = 27.5°, θ_min_ = 2.4°
φ and ω scans	*h* = −14→12
8492 measured reflections	*k* = −9→10
2812 independent reflections	*l* = −11→34

### Refinement

**Table:**

Refinement on *F*^2^	Primary atom site location: structure-invariant direct methods
Least-squares matrix: full	Secondary atom site location: difference Fourier map
*R*[*F*^2^ > 2σ(*F*^2^)] = 0.063	Hydrogen site location: inferred from neighbouring sites
*wR*(*F*^2^) = 0.146	H-atom parameters constrained
*S* = 1.02	*w* = 1/[σ^2^(*F*_o_^2^) + (0.0571*P*)^2^] where *P* = (*F*_o_^2^ + 2*F*_c_^2^)/3
2812 reflections	(Δ/σ)_max_ = 0.001
163 parameters	Δρ_max_ = 0.17 e Å^−^^3^
0 restraints	Δρ_min_ = −0.23 e Å^−^^3^

### Special details

**Table:**

Geometry. All esds (except the esd in the dihedral angle between two l.s. planes) are estimated using the full covariance matrix. The cell esds are taken into account individually in the estimation of esds in distances, angles and torsion angles; correlations between esds in cell parameters are only used when they are defined by crystal symmetry. An approximate (isotropic) treatment of cell esds is used for estimating esds involving l.s. planes.
Refinement. Refinement of *F*^2^ against ALL reflections. The weighted *R*-factor wR and goodness of fit *S* are based on *F*^2^, conventional *R*-factors *R* are based on *F*, with *F* set to zero for negative *F*^2^. The threshold expression of *F*^2^ > σ(*F*^2^) is used only for calculating *R*-factors(gt) etc. and is not relevant to the choice of reflections for refinement. *R*-factors based on *F*^2^ are statistically about twice as large as those based on *F*, and *R*- factors based on ALL data will be even larger.

### Fractional atomic coordinates and isotropic or equivalent isotropic displacement parameters (Å^2^)

**Table:**

	*x*	*y*	*z*	*U*_iso_*/*U*_eq_	
C1	0.88182 (18)	0.2124 (2)	0.28194 (7)	0.0385 (5)	
C2	0.78099 (18)	0.1252 (3)	0.26709 (8)	0.0441 (5)	
H2	0.7296	0.0839	0.2909	0.053*	
C3	0.75854 (19)	0.1013 (3)	0.21809 (8)	0.0462 (6)	
H3	0.6915	0.0437	0.2087	0.055*	
C4	0.83522 (18)	0.1623 (2)	0.18105 (7)	0.0373 (5)	
C5	0.8135 (2)	0.1425 (3)	0.12958 (8)	0.0479 (6)	
H5	0.7458	0.0881	0.1192	0.057*	
C6	0.8896 (2)	0.2014 (3)	0.09503 (8)	0.0486 (6)	
H6	0.8733	0.1879	0.0614	0.058*	
C7	0.99257 (19)	0.2824 (2)	0.10979 (7)	0.0410 (5)	
C8	1.01698 (18)	0.3049 (2)	0.15926 (7)	0.0386 (5)	
H8	1.0857	0.3584	0.1688	0.046*	
C9	0.93755 (18)	0.2466 (2)	0.19607 (7)	0.0349 (5)	
C10	0.95915 (17)	0.2723 (2)	0.24742 (7)	0.0356 (5)	
H10	1.0256	0.3296	0.2576	0.043*	
C11	0.9945 (2)	0.3094 (3)	0.35131 (8)	0.0511 (6)	
H11A	0.9876	0.4270	0.3460	0.061*	
H11B	1.0642	0.2707	0.3342	0.061*	
C12	1.0032 (2)	0.2742 (3)	0.40422 (8)	0.0538 (6)	
C13	1.0111 (2)	0.2413 (3)	0.44607 (10)	0.0777 (9)	
H13	1.0173	0.2149	0.4796	0.093*	
C14	1.1715 (2)	0.4069 (3)	0.08303 (7)	0.0486 (6)	
H14A	1.2248	0.3255	0.0967	0.058*	
H14B	1.1602	0.4926	0.1077	0.058*	
C15	1.2208 (2)	0.4766 (3)	0.03774 (8)	0.0512 (6)	
C16	1.2596 (2)	0.5391 (3)	0.00261 (10)	0.0727 (8)	
H16	1.2907	0.5892	−0.0256	0.087*	
O1	0.89297 (13)	0.22760 (17)	0.33268 (5)	0.0490 (4)	
O2	1.06171 (14)	0.33243 (19)	0.07121 (5)	0.0513 (4)	

### Atomic displacement parameters (Å^2^)

**Table:**

	*U*^11^	*U*^22^	*U*^33^	*U*^12^	*U*^13^	*U*^23^
C1	0.0421 (13)	0.0378 (11)	0.0356 (11)	0.0007 (10)	−0.0013 (9)	0.0027 (9)
C2	0.0396 (13)	0.0481 (13)	0.0448 (13)	−0.0091 (10)	0.0048 (10)	0.0058 (10)
C3	0.0381 (13)	0.0467 (13)	0.0540 (14)	−0.0078 (10)	−0.0041 (10)	−0.0019 (11)
C4	0.0371 (13)	0.0350 (11)	0.0398 (11)	0.0014 (9)	−0.0048 (9)	−0.0021 (9)
C5	0.0450 (14)	0.0507 (13)	0.0479 (13)	−0.0044 (11)	−0.0123 (11)	−0.0066 (11)
C6	0.0592 (16)	0.0554 (14)	0.0312 (11)	0.0003 (12)	−0.0104 (10)	−0.0058 (10)
C7	0.0487 (14)	0.0408 (12)	0.0335 (11)	0.0012 (10)	−0.0006 (10)	−0.0022 (9)
C8	0.0406 (13)	0.0422 (12)	0.0329 (11)	−0.0031 (10)	−0.0035 (9)	−0.0013 (9)
C9	0.0390 (12)	0.0324 (10)	0.0331 (10)	0.0024 (9)	−0.0026 (9)	−0.0015 (8)
C10	0.0357 (12)	0.0377 (11)	0.0333 (10)	−0.0018 (9)	−0.0032 (8)	0.0012 (8)
C11	0.0502 (15)	0.0653 (15)	0.0377 (12)	−0.0113 (12)	0.0010 (10)	0.0041 (10)
C12	0.0550 (16)	0.0705 (16)	0.0358 (12)	−0.0158 (13)	0.0011 (11)	−0.0001 (11)
C13	0.078 (2)	0.119 (2)	0.0368 (14)	−0.0317 (17)	−0.0031 (13)	0.0089 (14)
C14	0.0537 (15)	0.0559 (14)	0.0361 (11)	−0.0011 (12)	0.0023 (10)	−0.0015 (10)
C15	0.0618 (16)	0.0499 (14)	0.0420 (13)	0.0013 (12)	0.0130 (11)	−0.0047 (11)
C16	0.092 (2)	0.0675 (17)	0.0582 (17)	−0.0010 (15)	0.0295 (15)	0.0021 (14)
O1	0.0529 (10)	0.0647 (10)	0.0295 (8)	−0.0162 (8)	0.0016 (7)	0.0032 (7)
O2	0.0597 (10)	0.0663 (10)	0.0278 (8)	−0.0085 (9)	0.0018 (7)	−0.0025 (7)

### Geometric parameters (Å, °)

**Table:**

C1—C10	1.368 (3)	C8—H8	0.9300
C1—O1	1.375 (2)	C9—C10	1.417 (3)
C1—C2	1.406 (3)	C10—H10	0.9300
C2—C3	1.356 (3)	C11—O1	1.424 (2)
C2—H2	0.9300	C11—C12	1.454 (3)
C3—C4	1.414 (3)	C11—H11A	0.9700
C3—H3	0.9300	C11—H11B	0.9700
C4—C9	1.410 (3)	C12—C13	1.160 (3)
C4—C5	1.415 (3)	C13—H13	0.9300
C5—C6	1.357 (3)	C14—O2	1.424 (3)
C5—H5	0.9300	C14—C15	1.455 (3)
C6—C7	1.401 (3)	C14—H14A	0.9700
C6—H6	0.9300	C14—H14B	0.9700
C7—O2	1.364 (2)	C15—C16	1.160 (3)
C7—C8	1.371 (3)	C16—H16	0.9300
C8—C9	1.421 (3)		
			
C10—C1—O1	125.53 (18)	C4—C9—C10	119.59 (18)
C10—C1—C2	120.75 (19)	C4—C9—C8	119.19 (17)
O1—C1—C2	113.71 (17)	C10—C9—C8	121.22 (19)
C3—C2—C1	120.12 (19)	C1—C10—C9	119.76 (19)
C3—C2—H2	119.9	C1—C10—H10	120.1
C1—C2—H2	119.9	C9—C10—H10	120.1
C2—C3—C4	121.2 (2)	O1—C11—C12	107.91 (17)
C2—C3—H3	119.4	O1—C11—H11A	110.1
C4—C3—H3	119.4	C12—C11—H11A	110.1
C9—C4—C3	118.59 (18)	O1—C11—H11B	110.1
C9—C4—C5	118.69 (18)	C12—C11—H11B	110.1
C3—C4—C5	122.72 (19)	H11A—C11—H11B	108.4
C6—C5—C4	121.1 (2)	C13—C12—C11	177.9 (3)
C6—C5—H5	119.4	C12—C13—H13	180.0
C4—C5—H5	119.4	O2—C14—C15	108.50 (17)
C5—C6—C7	120.38 (19)	O2—C14—H14A	110.0
C5—C6—H6	119.8	C15—C14—H14A	110.0
C7—C6—H6	119.8	O2—C14—H14B	110.0
O2—C7—C8	125.5 (2)	C15—C14—H14B	110.0
O2—C7—C6	114.03 (18)	H14A—C14—H14B	108.4
C8—C7—C6	120.45 (19)	C16—C15—C14	176.9 (3)
C7—C8—C9	120.13 (19)	C15—C16—H16	180.0
C7—C8—H8	119.9	C1—O1—C11	117.76 (15)
C9—C8—H8	119.9	C7—O2—C14	117.59 (15)
			
C10—C1—C2—C3	−0.8 (3)	C3—C4—C9—C8	178.38 (18)
O1—C1—C2—C3	179.76 (19)	C5—C4—C9—C8	−1.7 (3)
C1—C2—C3—C4	0.2 (3)	C7—C8—C9—C4	1.5 (3)
C2—C3—C4—C9	1.2 (3)	C7—C8—C9—C10	−178.24 (18)
C2—C3—C4—C5	−178.8 (2)	O1—C1—C10—C9	179.43 (17)
C9—C4—C5—C6	0.7 (3)	C2—C1—C10—C9	0.1 (3)
C3—C4—C5—C6	−179.4 (2)	C4—C9—C10—C1	1.3 (3)
C4—C5—C6—C7	0.6 (3)	C8—C9—C10—C1	−179.01 (18)
C5—C6—C7—O2	178.94 (19)	C10—C1—O1—C11	−1.4 (3)
C5—C6—C7—C8	−0.8 (3)	C2—C1—O1—C11	178.01 (17)
O2—C7—C8—C9	−179.92 (18)	C12—C11—O1—C1	−165.49 (18)
C6—C7—C8—C9	−0.2 (3)	C8—C7—O2—C14	3.4 (3)
C3—C4—C9—C10	−1.9 (3)	C6—C7—O2—C14	−176.27 (18)
C5—C4—C9—C10	178.05 (18)	C15—C14—O2—C7	−170.02 (17)

### Hydrogen-bond geometry (Å, °)

**Table:**

Cg2 is the centroid of the C4–C9 benzene ring.

**Table:**

*D*—H···*A*	*D*—H	H···*A*	*D*···*A*	*D*—H···*A*
C13—H13···O2^i^	0.93	2.54	3.465 (3)	172
C14—H14B···O1^ii^	0.97	2.57	3.533 (3)	173
C11—H11A···Cg2^ii^	0.97	2.67	3.526 (3)	148

Symmetry codes: (i) *x*, −*y*+1/2, *z*+1/2; (ii) −*x*+2, *y*+1/2, −*z*+1/2.
